# Supplementary material for: CPT1A Supports Castration-Resistant Prostate Cancer in Androgen-Deprived Conditions
Source: Cells. 2019 Sep 20;8(10):1115. doi: 10.3390/cells8101115 (PMC6830347; doi:10.3390/cells8101115)
Supplement: Supplementary file 1 [file cells-08-01115-s001.pdf]

Supplementary tables, methods and figures

Supplementary Tables:

Supplementary Table 1: Primers

Supplementary Table 2: Primers		
**name**	**sequence**	**length**
AR (all forms)-F	GAAAGCGACTTCACCGCAC	20
AR (all forms)-R	AAAACATGGTCCCTGGCAGT	21
ARFL-F	ACATCAAGGAACTCGATCGTATC	23
ARFL-R	TTGGGCACTTGCACAGAGAT	20
ARv7-F	CCATCTTGTCGTCTTCGGAAATG	23
ARv7-R	TTTGAATGAGGCAAGTCAGCCTT	23
CPT1A-F	TGGATCTGCTGTATATCCTTC	21
CPT1A-R	AATTGGTTTGATTTCCTCCC	20
PSA-F	CGGATGCTGTGAAGGTCATGGA	22
PSA-R	GGGTCAAGAACTCCTCTGGTTC	21
RPL13A-F	CCTGGAGGAGAAGAGGAAAGAGA	23
RPL13A-R	TTGAGGACCTCTGTGTATTTGTCAA	25

Primers for NKX3.1, KRT19, DBI, DHCR24, ELOVL5 were purchased from Sigma Aldrich as pre-designed KiCqStart® SYBR® Green Primers.

Supplementary Table 2: Antibodies

**Supplementary Table 1: Antibodies**		
**Antibody**	**Catalogue number**	**Company**
GAPDH	5174	Cell Signaling
CPT1A	15184-1-AP	Proteintech
Acetylated Lysine	9441	Cell Signaling
H3K9ac	9649	Cell Signaling
H3K14ac	7627	Cell Signaling
H3K18ac	13998	Cell Signaling
H3K27ac	8137	Cell Signaling
H3	4499	Cell Signaling
H4K8ac	2594	Cell Signaling
H4	2935	Cell Signaling
H2AK5a	2576	Cell Signaling
H2A	12349	Cell Signaling
H2BK5ac	12799	Cell Signaling
H2B	12364	Cell Signaling

Supplementary Methods

Mass spectrometric analysis

Excised histone gel pieces were destained in ammonium bicarbonate/50% acetonitrile (ACN) and dehydrated in 100% acetonitrile. Disulfide bonds were reduced by dithiothreitol, and cysteine residues were alkylated with iodoacetamide. Sequencing grade Arg-C (Promega) was added 1:100 w/w and the samples were digested overnight at 37 C. The digestion was stopped by addition of 5% formic acid. Organic solvent was removed in a SpeedVac concentrator. The peptide mixture was desalted and concentrated on Thermo Scientific Pierce C18 Tip

Samples were analyzed on a Q Exactive quadrupole orbitrap mass spectrometer (Thermo Fisher Scientific, Waltham, MA, USA) coupled to an Easy nLC 1000 UHPLC (Thermo Fisher Scientific) through a nanoelectrospray ion source. Peptides were separated on a self-made 20 cm C18 analytical column (100 µm x 10 cm) packed with 2.7 µm Cortecs C18 resin. After equilibration with 3µL 5% acetonitrile 0.1% formic acid, the peptides were separated by a 120 min a linear gradient from 4% to 30% acetonitrile with 0.1% formic acid at 400nL/min. LC mobile phase solvents and sample dilutions used 0.1% formic acid in water (Buffer A) and 0.1% formic acid in acetonitrile (Buffer B) (Chromasolv LC–MS grade; Sigma-Aldrich, St. Louis, MO). Data acquisition was performed using the instrument supplied Xcalibur™ (version 3.0) software. The mass spectrometer was operated in the positive ion mode, in the data–dependent acquisition mode. The full MS scans were obtained with a range of m/z 300 to 1800, a mass resolution of 60,000 at m/z 200, and a target value of 1.00E+06 with the maximum injection time of 50 ms. HCD collision was performed on the 15 most significant peaks, and tandem mass spectra were acquired at a mass resolution of 15,000 at m/z 200 and a target value of 1.00E+05 the maximum injection time of 100 ms. Isolation of precursors was performed with a window of 2 Th. The dynamic exclusion time was 20s. The normalized collision energy was 30.

MS/MS spectra were extracted from raw data files and converted into mgf files *using Proteome Discoverer* 2.2. These mgf files were then independently searched against the human uniprotKB database (release date 2019.05) using an in-house Mascot™ server (Version 2.6, Matrix Science). Mass tolerances were +/- 10 ppm for MS peaks, and +/- 25ppm for MS/MS fragment ions. Arg-C specificity was used allowing for 1 missed cleavage. Met oxidation, protein N-terminal acetylation, peptide N-terminal pyroglutamic acid formation, C^{13} acetyl on lysine, acetylation on lysine, methylation on lysine and arginine, dimethylation on lysine and arginine, and Cys carbamido methylation were allowed for variable modifications.

Scaffold (version 4.8, Proteome Software, Portland, OR, USA) was used to validate MS/MS based peptide and protein identifications. Peptide identifications were accepted if they could be established at greater than 95.0% probability as specified by the Peptide Prophet algorithm. Protein identifications were accepted if they could be established at greater than 99.0% probability and contained at least two identified unique peptides.

Supplementary Figures

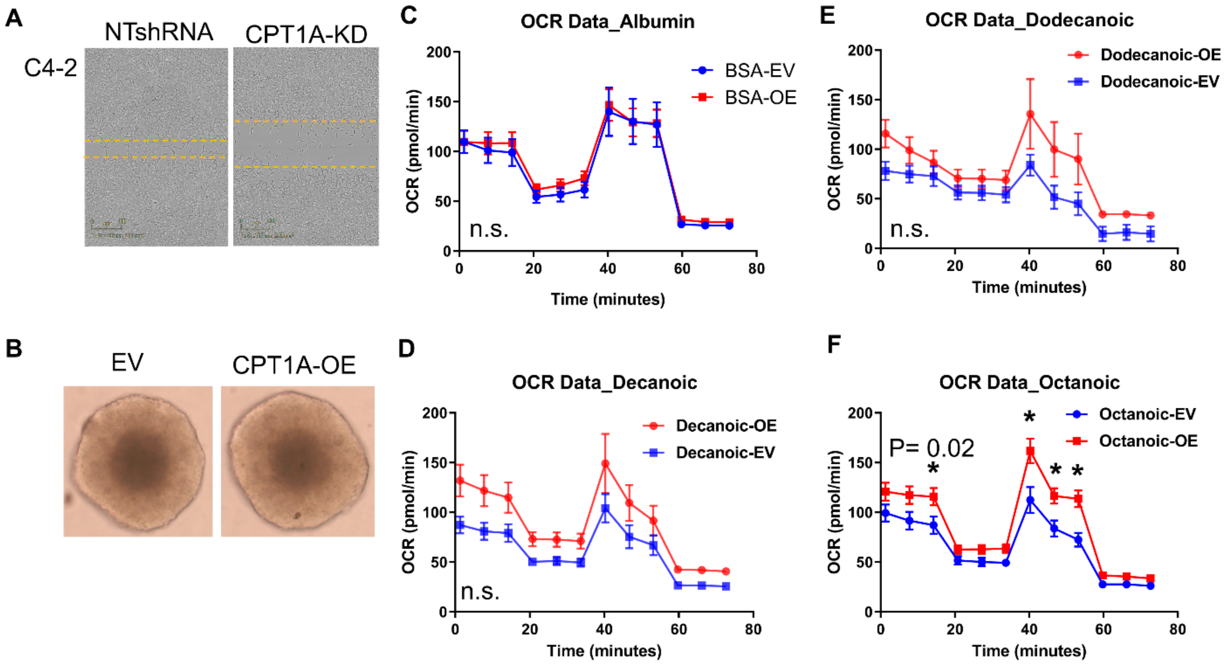

Supplementary Figure 1: Supplementary data for Figures 1 and 2 characterizing the C4-2 CPT1A-KD and CPT1A-OE cells.

A) Representative image from the scratch wound / migration assay with C4-2 CPT1A-KD and control cells. B) Representative images of spheroid assay with C42 CPT1A-OE and control EV cells, n= 8 each. C) Oxygen Consumption Rate (OCR) of C4-2 CPT1A-OE and control empty vector EV in the presence of dodecanoic, decanoic and octanoic fatty acids conjugated to BSA. OCR was measured using the Seahorse XFe96 Analyzer. Cells were serum starved overnight and then incubated with the BSA-conjugated fatty acids 1 hour before the assay. Traces shown correspond to Cell Mito Stress test using Oligomycin (2 uM), FCCP (2 uM) and rotenone/antimycin A (1 uM) at 20, 40 and 60 min respectively.

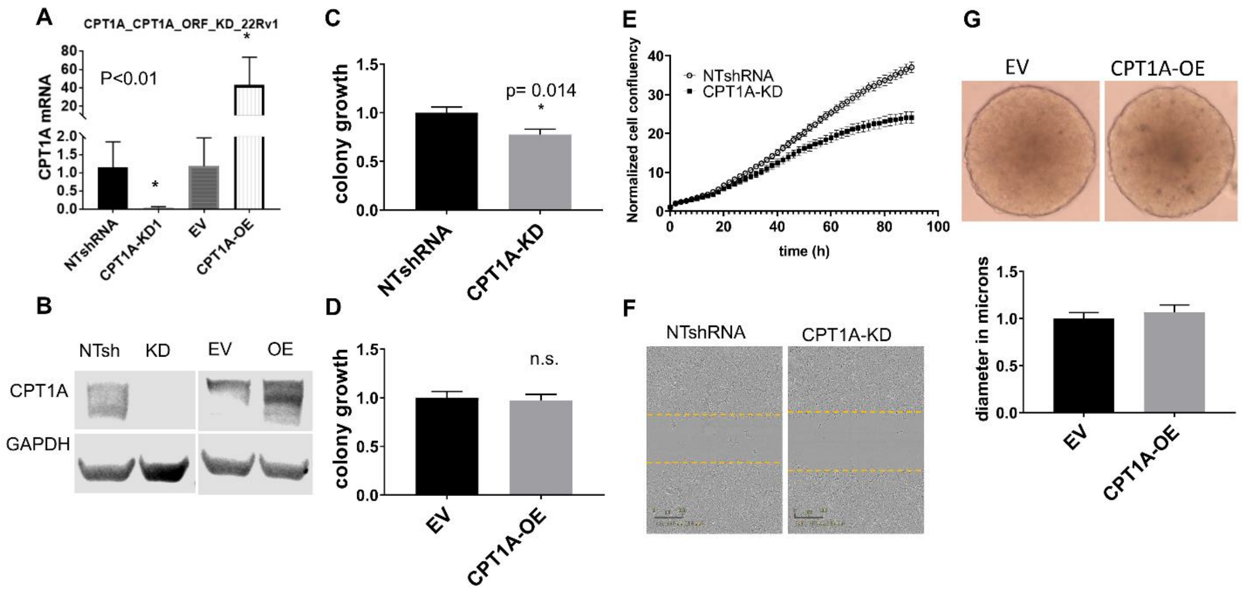

Supplementary figure 2: Growth and migration of 22Rv1 cells with CPT1A knockdown and overexpression.

A) CPT1A mRNA levels were measured in CPT1A knockdown (KD) and overexpressed (OE) 22Rv1 cells using qRT-PCR. Each measurement shows the averaged value of 4 independent replicates, which were normalized to expression levels of RPL13A mRNA. B) Representative Western blot of CPT1A protein in KD and OE cells. C) Clonogenic growth of KD cells compared to control cells. D) Clonogenic growth of OE cells compared to their control cells. E-F) graph and representative image from the scratch wound / migration assay with 22Rv1 CPT1A-KD and control cells, n=10 per group. G) Representative images and bar graph of spheroid assay with C42 CPT1A-OE and control EV cells, n= 8 each(no significant).

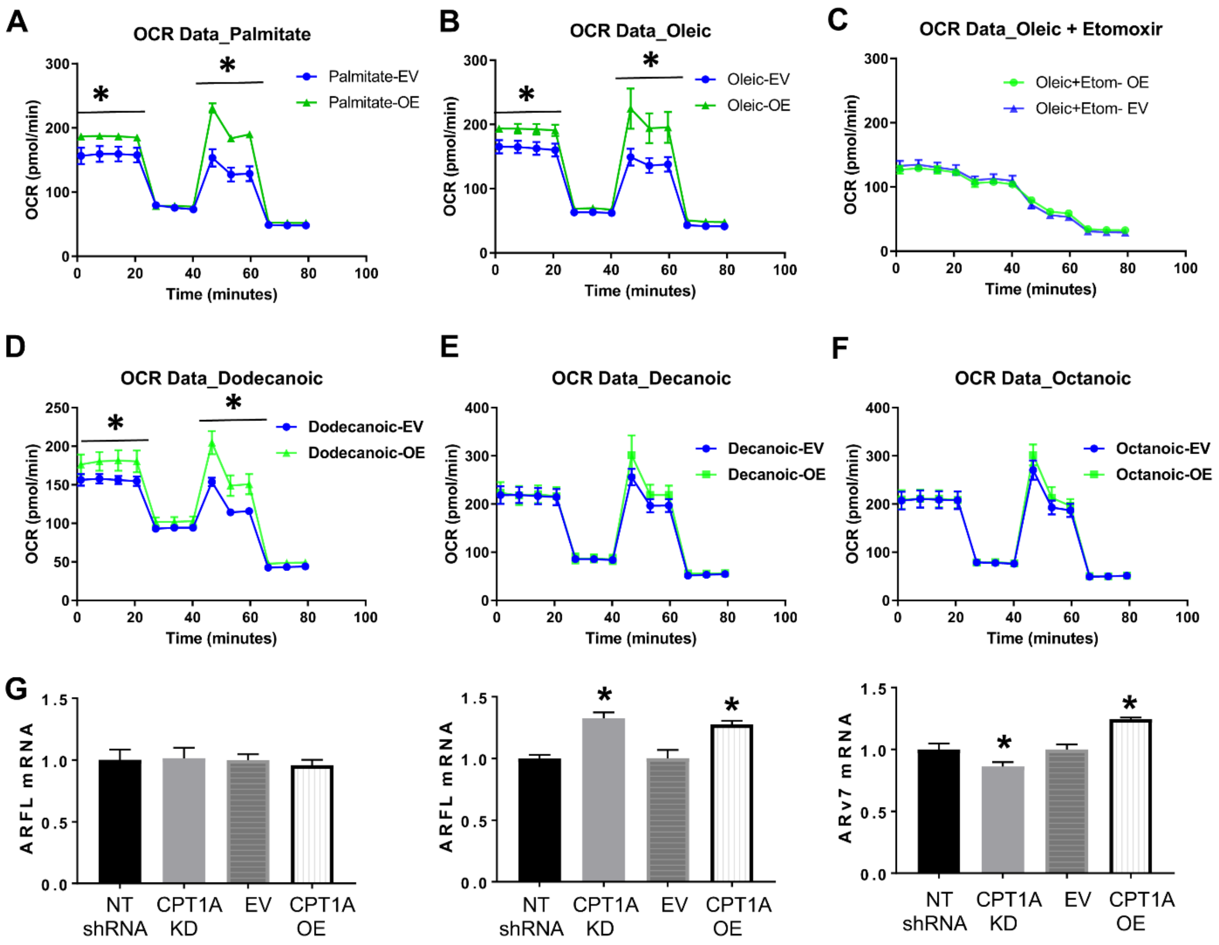

Supplementary figure 3: Seahorse OCR traces and AR isoforms expression in 22Rv1 cells with CPT1A knockdown and overexpression.

A-F) Oxygen Consumption Rates (OCR) of 22Rv1 CPT1A-OE and control empty vector (EV) in the presence of palmitate (A), oleic (B), oleic +etomoxir (C), dodecanoic(D), decanoic (E) and octanoic (F) were measured using the Seahorse XFe96 Analyzer. Cells were serum starved overnight and then incubated with the BSA-conjugated fatty acids 1 hour before the assay Etomoxir (40uM) was added to the assay as a control to block fat oxidation. Panel Traces shown correspond to Cell Mito Stress test using Oligomycin (2 uM), FCCP (2 uM) and rotenone/antimycin A (1 uM) at 20, 40 and 60 min respectively. G) AR expression in response to DHT in 22Rv1. Expression of AR-all (measures all isoforms of AR), ARFL (full length AR) and ARv7 (AR variant v7) was measured in response to 100pM DHT in CPT1A-OE and control EV cells, (n=4 each), $^*p \leq 0.01$ compared to its respective control. Statistical significance was calculated using ANOVA with Holm-Sidak method.

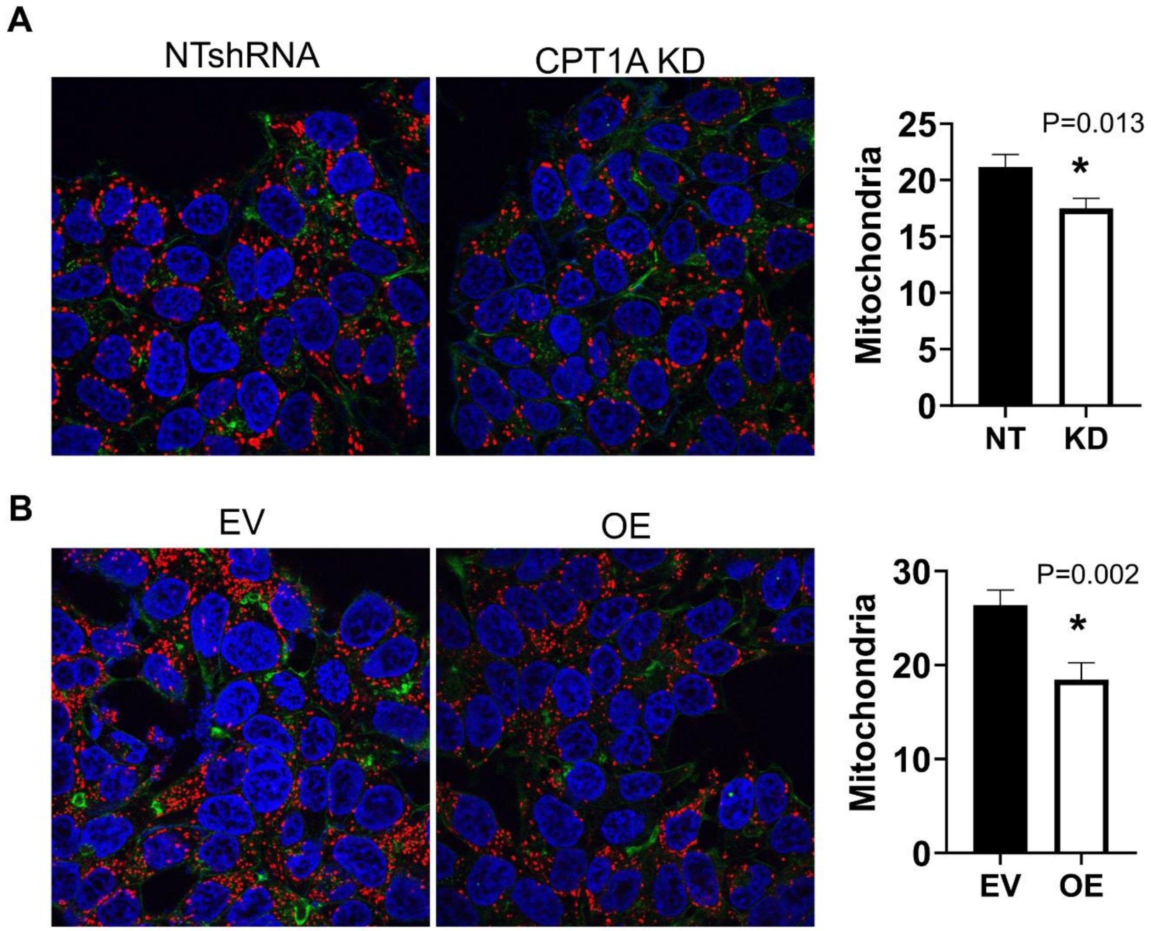

Supplementary figure 4: Mitochondrial content of 22Rv1 cells with CPT1A knockdown and overexpression.

A-B) Mitochondrial and lipid droplet staining in CPT1A-KD cells (A) and CPT1A-OE cells (B). Cells were stained for lipid droplets (green), mitochondria (red) and nuclei (blue). A minimum of 20 cells per group were captured and quantitated using ImageJ. Significance was calculated using Students t-test. We did not quantify the lipid droplets (green channel) due to the weak signal of the stain.

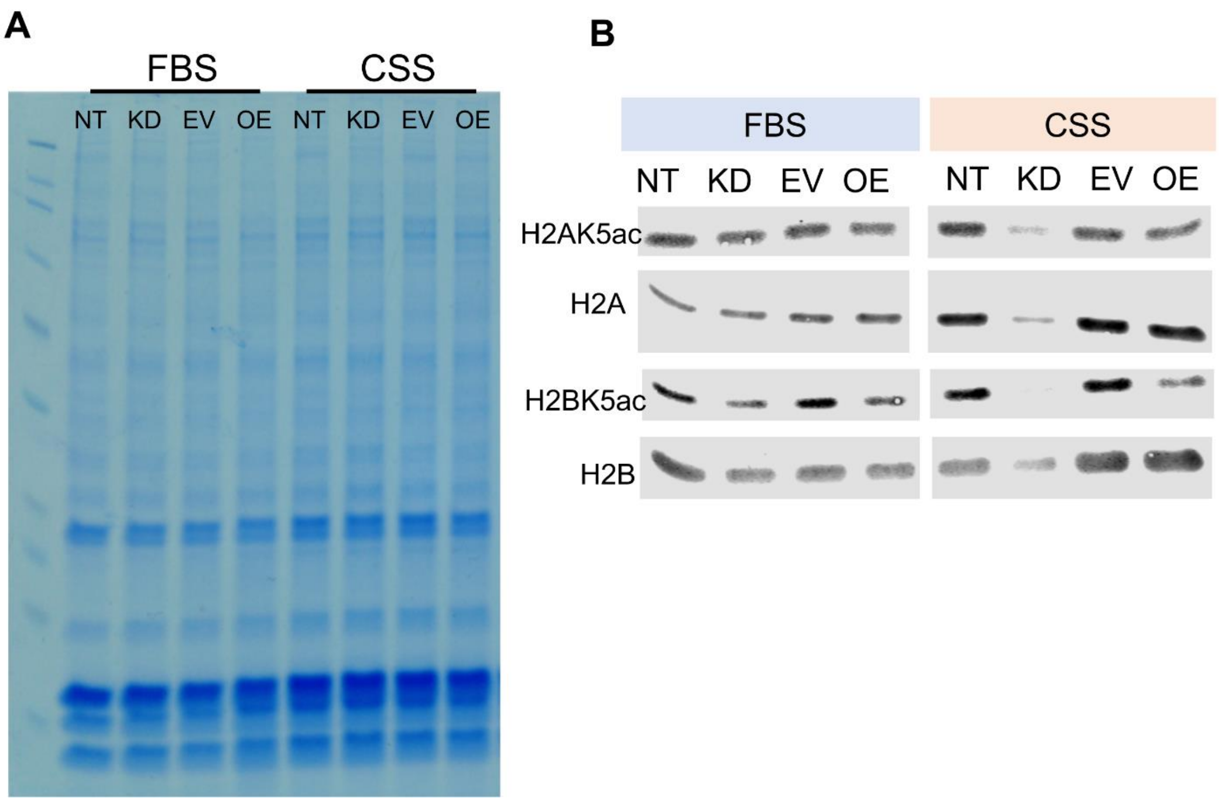

Supplementary figure 5: Supplementary data to Figure 6.

A) Coomassie stain of C4-2 isolated histones under FBS or CSS conditions. The isolated histones were used for mass spec analysis. B) Additional Western blots of acetylation marks in the CPT1A KD and OE C4-2 cell lysates under FBS or CSS conditions.
